# Supplementary material for: Proteomic Changes in the Human Cerebrovasculature in Alzheimer’s Disease and Related Tauopathies Linked to Peripheral Biomarkers in Plasma and Cerebrospinal Fluid
Source: medRxiv. 2024 Jan 11:2024.01.10.24301099. Preprint. [Version 1] doi: 10.1101/2024.01.10.24301099 (PMC10802758; doi:10.1101/2024.01.10.24301099)
Supplement: Supplement 2 [file NIHPP2024.01.10.24301099v1-supplement-2.pdf]

medRxiv preprint doi: https://doi.org/10.1101/2024.01.10.24301099; this version posted January 11, 2024. The copyright holder for this preprint (which was not certified by peer review) is the author/funder, who has granted medRxiv a license to display the preprint in perpetuity.
It is made available under a CC-BY 4.0 International license .

Supplemental Figure 1. Establishment of the method of cerebrovascular isolation (**A**) Label free mass spectrometry analysis of proteins enriched in whole brain, parenchyma, and isolated cerebrovasculature. Red indicates proteins increased and blue indicates proteins decreased in each brain fraction. (**B**) Western blot analysis shows enrichment of vascular-specific proteins in the vascular fractions compared to whole brain and parenchyma. (**C**) Microscopy images showing the purity of isolated vasculature (10x magnification).

Supplemental Figure 2. Cell type proportions in the vascular and bulk proteomes. (**A**) Heatmap showing cell type proportions in the vascular proteome (above dashed line) and bulk proteome (bottom). Proportions of the five cell type total one, and the five cell types are defined via reprocessing of Darmanis[79], Nowakowski[80], and Zhong single-cell datasets as curated with the EnsDeconv R package, which calculated cell type proportions by ensemble deconvolution of each proteome[81]. Dark red and orange indicate high enrichment of endothelial proteins to proportions above 70 percent, and dark blue indicates the lowest enrichment of cell-specific proteins in the preparations. The purple box outlines a cluster of outliers in the vascular preparations with very low enrichment of endothelial markers. (**B**) Variance partition plots were used to visualize the percent variance of each protein in the data set co-varying with batch, group, age, sex, postmortem interval (PMI), and neuronal proportions across case samples. (**C**) Following outlier removal, the matrix was subjected to the bootstrap regression to remove the variance due to age, sex, PMI, and neuronal cell type proportions.

Supplemental Figure 3. Differentially abundant proteins unique to vascular and bulk proteomes reveals specific discriminators. (A) Venn diagram of shared and unique proteins discovered in vascular and bulk fractions; counts shown are for unique gene products, in distinction from total protein isoforms. (**B**) GO terms enrichment analysis was used to represent biological processes, molecular function, and cellular

medRxiv preprint doi: https://doi.org/10.1101/2024.01.10.24301099; this version posted January 11, 2024. The copyright holder for this preprint (which was not certified by peer review) is the author/funder, who has granted medRxiv a license to display the preprint in perpetuity.
It is made available under a CC-BY 4.0 International license .

component for unique vascular or bulk proteins. **(C-D)** Proteins unique to either vascular fraction or bulk proteome were input for statistics underlying volcano plots displaying differential abundance of 582 proteins between Control and AD (**C**) or 321 proteins between Control and PSP (**D**). The x axis shows the log_2 fold change, while the y axis represents $-log_{10}$ statistical *p* value calculated for all proteins between pairwise vascular and bulk group comparisons in AD (**C**) and PSP (**D**). One-way ANOVA was performed followed by Tukey's post-hoc test for each pairwise comparison vs. control and imprecise Tukey p values below $10^{-8.5}$ were replaced with pairwise two-sided unequal variance T-tests' p values corrected for multiple tests by Bonferroni correction. Proteins are colored based on the proteome membership (red indicates vascular proteome and green indicates bulk proteome).

Supplemental Figure 4. Aβ40 is predominantly found in the brain vascular fraction. (**A**) MS-quantified levels of the C-terminal tryptic Aβ40 and Aβ42 species in the vascular and bulk fractions. (**B**) MS/MS spectrum of C-terminal tryptic peptide of Aβ42 with annotation of matched B and Y ions as indicated. (**C**) MS/MS spectrum of the cognate peptide for Aβ40.

Supplemental Figure 5. Amyloid-associated changes in cerebrovasculature drive distinct proteomic signatures in AD and PSP. (A) Volcano plot showing the log_2 fold change (x axis) and $-log_{10}$ one-way ANOVA with Tukey *p* value (y axis) for differentially changed proteins between pairwise AD and PSP comparison. Tukey *p* values below $10^{-8.5}$ were recalculated as Bonferroni-corrected two-tailed unequal variance t-test *p* values. Proteins are shaded based on their module membership colors. **(B)** Scatter plot of the cerebrovascular proteome log_2 effect size of the PSP group compared to unimpaired healthy Controls (x) versus the log_2 effect size of the AD group compared to Controls (y). Highlighted proteins showcased disease-specific signatures altered in AD and PSP.

Supplemental Table S1. Sample characteristics - vascular and bulk proteomes.

Supplemental Table S2. Cell type-specific marker list from snRNA-seq.

Supplemental Table S3. Protein differential abundance analysis of cerebrovasculature (ANOVA).

Supplemental Table S4. Protein differential abundance analysis of bulk tissue (ANOVA).

Supplemental Table S5. Cerebrovascular WGCNA module assignment.

Supplemental Table S6. Gene ontology terms for cerebrovascular protein modules.

Supplemental Table S7. The correlation analysis of cerebrovascular protein modules.

Supplemental Table S8. MAGMA GWAS significance for genes associated with AD.

Supplemental Table S9. MAGMA GWAS significance for genes associated with PSP.

Supplemental Table S10. Sample characteristics - plasma proteome.

medRxiv preprint doi: https://doi.org/10.1101/2024.01.10.24301099; this version posted January 11, 2024. The copyright holder for this preprint (which was not certified by peer review) is the author/funder, who has granted medRxiv a license to display the preprint in perpetuity.
It is made available under a CC-BY 4.0 International license .

Supplemental Table S11. Sample characteristics - CSF proteome.

Supplemental Table S12. Protein differential abundance of plasma (ANOVA).

Supplemental Table S13. Protein differential abundance of CSF (ANOVA).

Supplemental Table S14. Protein overlap between plasma and cerebrovascular datasets (FET analysis).

Supplemental Table S15. Protein overlap between CSF and cerebrovascular datasets (FET analysis).

medRxiv preprint doi: https://doi.org/10.1101/2024.01.10.24301099; this version posted January 11, 2024. The copyright holder for this preprint (which was not certified by peer review) is the author/funder, who has granted medRxiv a license to display the preprint in perpetuity.
It is made available under a CC-BY 4.0 International license .

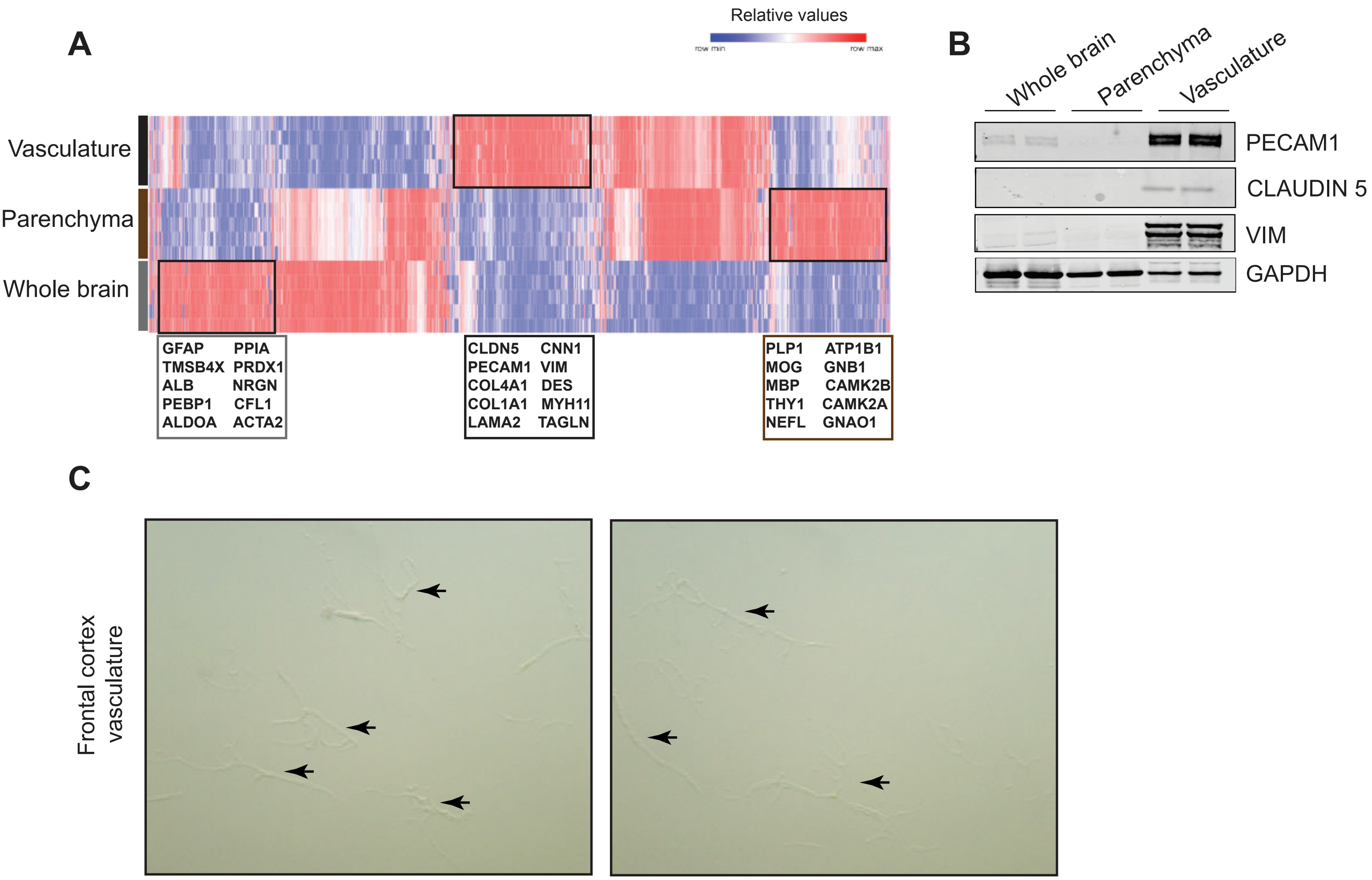

medRxiv preprint doi: https://doi.org/10.1101/2024.01.10.24301099; this version posted January 11, 2024. The copyright holder for this preprint
(which was not certified by peer review) is the author/funder, who has granted medRxiv a license to display the preprint in perpetuity.
It is made available under a CC-BY 4.0 International license .

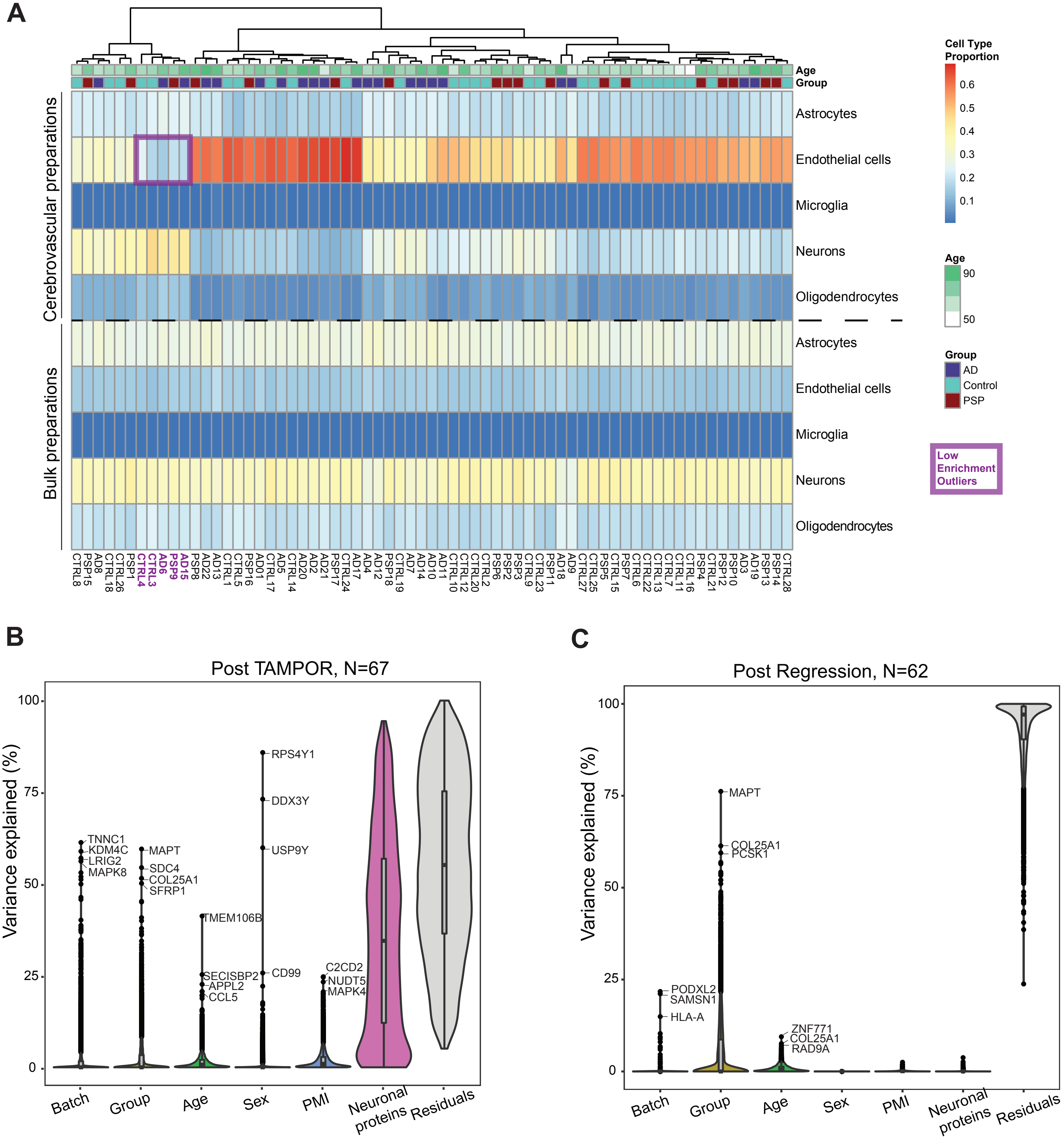

medRxiv preprint doi: https://doi.org/10.1101/2024.01.10.24301099; this version posted January 11, 2024. The copyright holder for this preprint (which was not certified by peer review) is the author/funder, who has granted medRxiv a license to display the preprint in perpetuity.
It is made available under a CC-BY 4.0 International license .

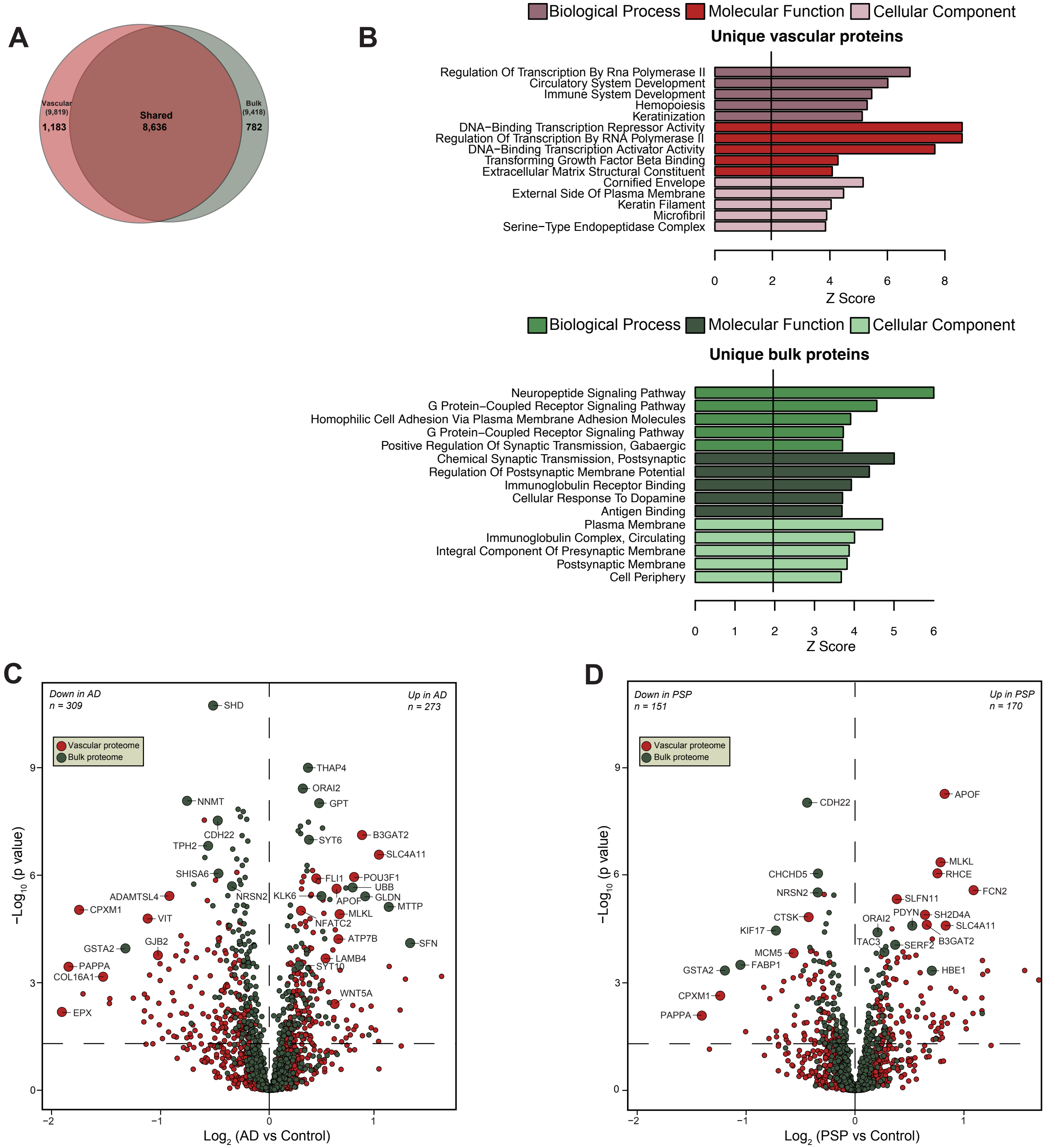

medRxiv preprint doi: https://doi.org/10.1101/2024.01.10.24301099; this version posted January 11, 2024. The copyright holder for this preprint (which was not certified by peer review) is the author/funder, who has granted medRxiv a license to display the preprint in perpetuity.
It is made available under a CC-BY 4.0 International license .

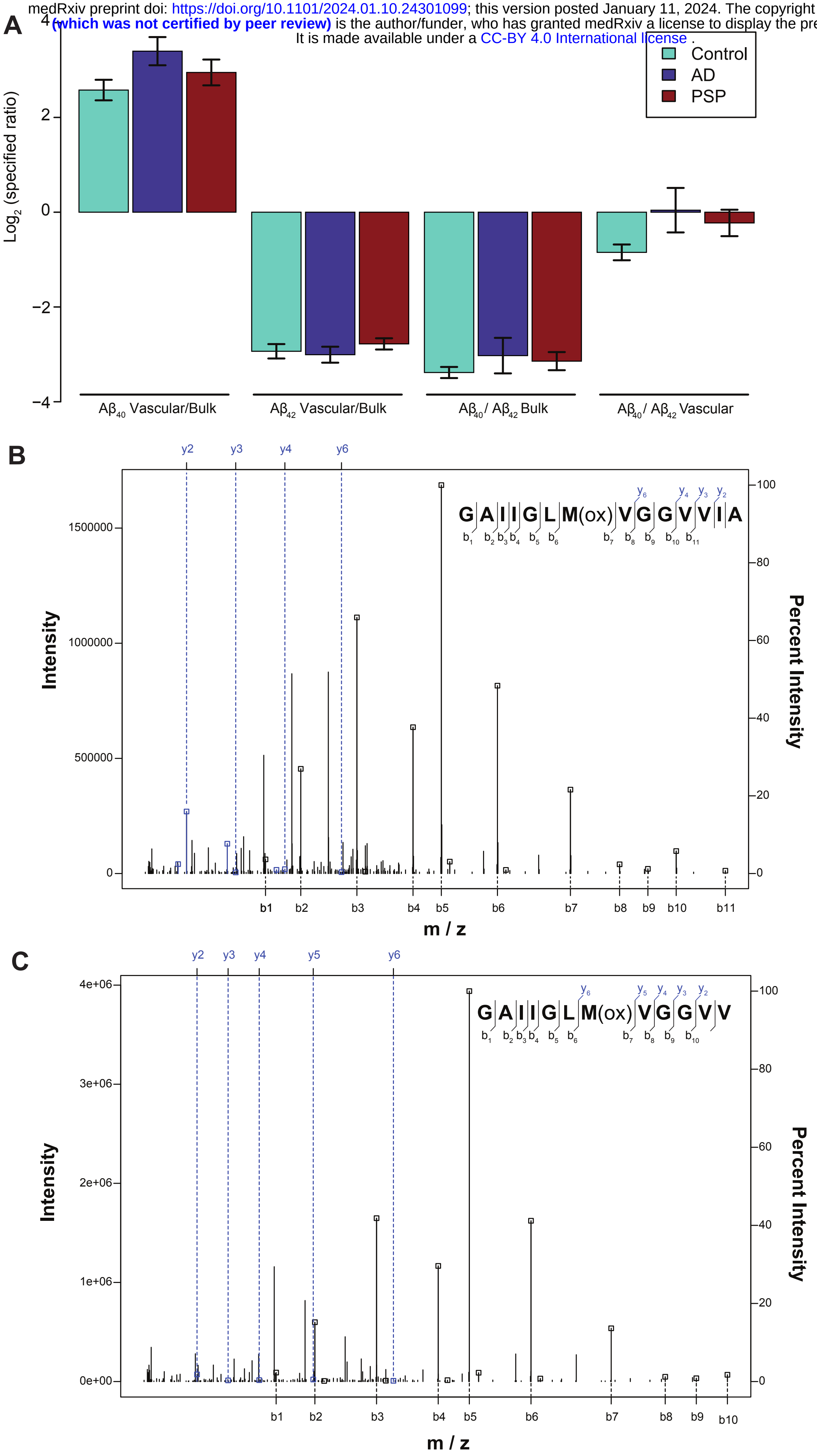

medRxiv preprint doi: https://doi.org/10.1101/2024.01.10.24301099; this version posted January 11, 2024. The copyright holder for this preprint (which was not certified by peer review) is the author/funder, who has granted medRxiv a license to display the preprint in perpetuity.
It is made available under a CC-BY 4.0 International license .

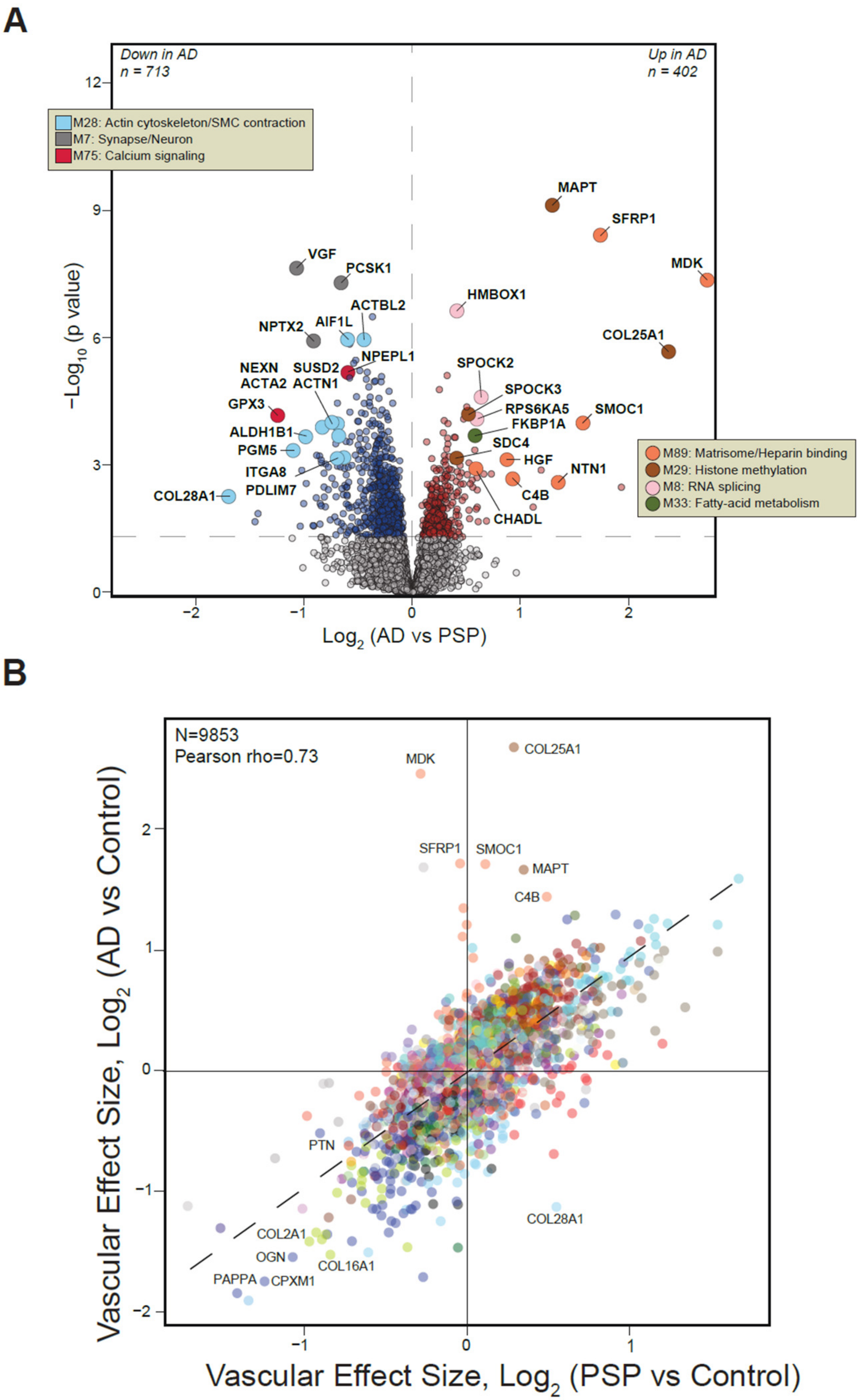

medRxiv preprint doi: https://doi.org/10.1101/2024.01.10.24301099; this version posted January 11, 2024. The copyright holder for this preprint (which was not certified by peer review) is the author/funder, who has granted medRxiv a license to display the preprint in perpetuity.
It is made available under a CC-BY 4.0 International license .

**Sample Size**	Control<br>*N*= 28	AD<br>*N*= 21	PSP<br>*N*= 18
**Characteristics**			
**Age**, *years± SD*	65.8 ± 8.0	78.8 ± 10.5	76.3 ± 5.5
**Sex**, *N Males*	15	9	11
N Females	13	12	7
**PMI**, *years± SD*	14.6 ± 5.3	12.2 ± 5.4	11.6 ± 6.2
**Amyloid burden**, *CERAD 0-1*	27	0	17
CERAD 2-3	1	21	1
**Tau burden**, *Braak 0-I*	28	0	9
Braak, II-III	0	21	9
**CAA score**, *0 - no CAA*	25	2	14
1 - mild CAA	2	7	3
2 - moderate CAA	0	5	1
3 - severe CAA	1	7	0
***APOE* genotype**, *E44*	1	6	0
E34	5	4	2
E33	19	11	9
E24	2	0	0
E23	1	0	5

Abbreviations: Ctrl, Control; AD, Alzheimer's disease; PSP, progressive supranuclear palsy; M, males; F, females; PMI, postmortem interval; APOE, apolipoprotein E

medRxiv preprint doi: https://doi.org/10.1101/2024.01.10.24301099; this version posted January 11, 2024. The copyright holder for this preprint (which was not certified by peer review) is the author/funder, who has granted medRxiv a license to display the preprint in perpetuity.
It is made available under a CC-BY 4.0 International license .

**Sample Size**	Ctrl<br>*N*= 141	AD<br>*N*= 140
**Characteristics**		
**Race,** *African American*	18	10
Caucasian	122	129
Other	1	1
**Age**, *years± SD*	64.7 ± 7.8	68.1 ± 8.4
**Sex**, *N Males*	37	65
N Females	104	75
***APOE* genotype**, *E44*	3	28
E34	29	71
E33	75	38
E24	7	2
E23	25	1

Abbreviations: Ctrl, Control; AD, Alzheimer's disease; M, males; F, females; APOE, apolipoprotein E
